# Supplementary material for: Pyruvate Dehydrogenase Kinase 1 inhibition mediated oxidative phosphorylation enhancement in cartilage promotes osteoarthritis progression
Source: BMC Musculoskelet Disord. 2023 Jul 20;24:597. doi: 10.1186/s12891-023-06585-6 (PMC10357736; doi:10.1186/s12891-023-06585-6)
Supplement: Supplementary file 1 — Additional file 1. [file 12891_2023_6585_MOESM1_ESM.zip › BP.Gsea.1653623667859/GOBP_GOLGI_VESICLE_TRANSPORT.html]

Details for gene set GOBP\_GOLGI\_VESICLE\_TRANSPORT[GSEA]

|  || Dataset | 55457\_collapsed\_to\_symbols.cls.cls #OA\_versus\_normal.cls.cls #OA\_versus\_normal\_repos |
| Phenotype | cls.cls#OA\_versus\_normal\_repos |
| Upregulated in class | OA |
| GeneSet | GOBP\_GOLGI\_VESICLE\_TRANSPORT |
| Enrichment Score (ES) | 0.32840183 |
| Normalized Enrichment Score (NES) | 1.7609047 |
| Nominal p-value | 0.0 |
| FDR q-value | 0.10262592 |
| FWER p-Value | 0.998 |
Table: GSEA Results Summary

  

Fig 1: Enrichment plot: GOBP\_GOLGI\_VESICLE\_TRANSPORT      
 Profile of the Running ES Score & Positions of GeneSet Members on the Rank Ordered List

  

| SYMBOL | TITLE | RANK IN GENE LIST | RANK METRIC SCORE | RUNNING ES | CORE ENRICHMENT || 1 | COPE | COPI coat complex subunit epsilon [Source:HGNC Symbol;Acc:HGNC:2234] | 15 | 1.136 | 0.0143 | Yes |
| 2 | COPZ2 | COPI coat complex subunit zeta 2 [Source:HGNC Symbol;Acc:HGNC:19356] | 19 | 1.109 | 0.0291 | Yes |
| 3 | COG5 | component of oligomeric golgi complex 5 [Source:HGNC Symbol;Acc:HGNC:14857] | 38 | 1.012 | 0.0414 | Yes |
| 4 | SNX2 | sorting nexin 2 [Source:HGNC Symbol;Acc:HGNC:11173] | 43 | 1.007 | 0.0548 | Yes |
| 5 | KDELR3 | KDEL endoplasmic reticulum protein retention receptor 3 [Source:HGNC Symbol;Acc:HGNC:6306] | 77 | 0.950 | 0.0651 | Yes |
| 6 | PDCD6 | programmed cell death 6 [Source:HGNC Symbol;Acc:HGNC:8765] | 87 | 0.933 | 0.0771 | Yes |
| 7 | ARL1 | ADP ribosylation factor like GTPase 1 [Source:HGNC Symbol;Acc:HGNC:692] | 100 | 0.914 | 0.0886 | Yes |
| 8 | YIPF5 | Yip1 domain family member 5 [Source:HGNC Symbol;Acc:HGNC:24877] | 107 | 0.903 | 0.1004 | Yes |
| 9 | TMED3 | transmembrane p24 trafficking protein 3 [Source:HGNC Symbol;Acc:HGNC:28889] | 141 | 0.868 | 0.1096 | Yes |
| 10 | YIF1A | "Yip1 interacting factor homolog A, membrane trafficking protein [Source:HGNC Symbol;Acc:HGNC:16688]" | 145 | 0.867 | 0.1211 | Yes |
| 11 | ERGIC3 | ERGIC and golgi 3 [Source:HGNC Symbol;Acc:HGNC:15927] | 299 | 0.751 | 0.1192 | Yes |
| 12 | TRAPPC4 | trafficking protein particle complex subunit 4 [Source:HGNC Symbol;Acc:HGNC:19943] | 322 | 0.736 | 0.1275 | Yes |
| 13 | VTI1B | vesicle transport through interaction with t-SNAREs 1B [Source:HGNC Symbol;Acc:HGNC:17793] | 336 | 0.732 | 0.1364 | Yes |
| 14 | RAB8A | "RAB8A, member RAS oncogene family [Source:HGNC Symbol;Acc:HGNC:7007]" | 383 | 0.712 | 0.1425 | Yes |
| 15 | COPZ1 | COPI coat complex subunit zeta 1 [Source:HGNC Symbol;Acc:HGNC:2243] | 421 | 0.697 | 0.1490 | Yes |
| 16 | SEC13 | "SEC13 homolog, nuclear pore and COPII coat complex component [Source:HGNC Symbol;Acc:HGNC:10697]" | 460 | 0.677 | 0.1552 | Yes |
| 17 | LMAN2 | "lectin, mannose binding 2 [Source:HGNC Symbol;Acc:HGNC:16986]" | 485 | 0.666 | 0.1624 | Yes |
| 18 | COPB2 | COPI coat complex subunit beta 2 [Source:HGNC Symbol;Acc:HGNC:2232] | 504 | 0.658 | 0.1699 | Yes |
| 19 | LMAN2L | "lectin, mannose binding 2 like [Source:HGNC Symbol;Acc:HGNC:19263]" | 507 | 0.656 | 0.1787 | Yes |
| 20 | GOLPH3L | golgi phosphoprotein 3 like [Source:HGNC Symbol;Acc:HGNC:24882] | 521 | 0.652 | 0.1865 | Yes |
| 21 | TRAPPC6A | trafficking protein particle complex subunit 6A [Source:HGNC Symbol;Acc:HGNC:23069] | 636 | 0.610 | 0.1858 | Yes |
| 22 | RANGRF | RAN guanine nucleotide release factor [Source:HGNC Symbol;Acc:HGNC:17679] | 660 | 0.603 | 0.1922 | Yes |
| 23 | RER1 | retention in endoplasmic reticulum sorting receptor 1 [Source:HGNC Symbol;Acc:HGNC:30309] | 724 | 0.588 | 0.1952 | Yes |
| 24 | GABARAPL2 | GABA type A receptor associated protein like 2 [Source:HGNC Symbol;Acc:HGNC:13291] | 776 | 0.575 | 0.1990 | Yes |
| 25 | AP3S2 | adaptor related protein complex 3 subunit sigma 2 [Source:HGNC Symbol;Acc:HGNC:571] | 780 | 0.574 | 0.2066 | Yes |
| 26 | PREPL | prolyl endopeptidase like [Source:HGNC Symbol;Acc:HGNC:30228] | 810 | 0.566 | 0.2120 | Yes |
| 27 | BNIP1 | BCL2 interacting protein 1 [Source:HGNC Symbol;Acc:HGNC:1082] | 824 | 0.563 | 0.2186 | Yes |
| 28 | NSF | "N-ethylmaleimide sensitive factor, vesicle fusing ATPase [Source:HGNC Symbol;Acc:HGNC:8016]" | 908 | 0.540 | 0.2194 | Yes |
| 29 | VPS51 | VPS51 subunit of GARP complex [Source:HGNC Symbol;Acc:HGNC:1172] | 911 | 0.539 | 0.2265 | Yes |
| 30 | TRAPPC2 | trafficking protein particle complex subunit 2 [Source:HGNC Symbol;Acc:HGNC:23068] | 914 | 0.538 | 0.2337 | Yes |
| 31 | TEX261 | testis expressed 261 [Source:HGNC Symbol;Acc:HGNC:30712] | 948 | 0.529 | 0.2383 | Yes |
| 32 | COG4 | component of oligomeric golgi complex 4 [Source:HGNC Symbol;Acc:HGNC:18620] | 966 | 0.525 | 0.2441 | Yes |
| 33 | TRAPPC2L | trafficking protein particle complex subunit 2L [Source:HGNC Symbol;Acc:HGNC:30887] | 990 | 0.520 | 0.2493 | Yes |
| 34 | GOLGA5 | golgin A5 [Source:HGNC Symbol;Acc:HGNC:4428] | 1016 | 0.514 | 0.2543 | Yes |
| 35 | DNAJC28 | DnaJ heat shock protein family (Hsp40) member C28 [Source:HGNC Symbol;Acc:HGNC:1297] | 1093 | 0.498 | 0.2551 | Yes |
| 36 | NBAS | NBAS subunit of NRZ tethering complex [Source:HGNC Symbol;Acc:HGNC:15625] | 1132 | 0.490 | 0.2588 | Yes |
| 37 | SEC22A | "SEC22 homolog A, vesicle trafficking protein [Source:HGNC Symbol;Acc:HGNC:20260]" | 1220 | 0.472 | 0.2583 | Yes |
| 38 | BET1 | Bet1 golgi vesicular membrane trafficking protein [Source:HGNC Symbol;Acc:HGNC:14562] | 1223 | 0.471 | 0.2646 | Yes |
| 39 | ARL3 | ADP ribosylation factor like GTPase 3 [Source:HGNC Symbol;Acc:HGNC:694] | 1264 | 0.465 | 0.2677 | Yes |
| 40 | KDELR2 | KDEL endoplasmic reticulum protein retention receptor 2 [Source:HGNC Symbol;Acc:HGNC:6305] | 1486 | 0.431 | 0.2561 | Yes |
| 41 | SPAST | spastin [Source:HGNC Symbol;Acc:HGNC:11233] | 1502 | 0.429 | 0.2608 | Yes |
| 42 | BCAP29 | B cell receptor associated protein 29 [Source:HGNC Symbol;Acc:HGNC:24131] | 1548 | 0.421 | 0.2629 | Yes |
| 43 | BBS1 | Bardet-Biedl syndrome 1 [Source:HGNC Symbol;Acc:HGNC:966] | 1556 | 0.419 | 0.2681 | Yes |
| 44 | ARF3 | ADP ribosylation factor 3 [Source:HGNC Symbol;Acc:HGNC:654] | 1572 | 0.418 | 0.2726 | Yes |
| 45 | BCAP31 | B cell receptor associated protein 31 [Source:HGNC Symbol;Acc:HGNC:16695] | 1584 | 0.416 | 0.2774 | Yes |
| 46 | RAB6A | "RAB6A, member RAS oncogene family [Source:HGNC Symbol;Acc:HGNC:9786]" | 1599 | 0.413 | 0.2819 | Yes |
| 47 | RAB2A | "RAB2A, member RAS oncogene family [Source:HGNC Symbol;Acc:HGNC:9763]" | 1633 | 0.409 | 0.2848 | Yes |
| 48 | MPPE1 | metallophosphoesterase 1 [Source:HGNC Symbol;Acc:HGNC:15988] | 1675 | 0.402 | 0.2871 | Yes |
| 49 | COG7 | component of oligomeric golgi complex 7 [Source:HGNC Symbol;Acc:HGNC:18622] | 1691 | 0.400 | 0.2913 | Yes |
| 50 | USE1 | unconventional SNARE in the ER 1 [Source:HGNC Symbol;Acc:HGNC:30882] | 1723 | 0.395 | 0.2942 | Yes |
| 51 | CCDC91 | coiled-coil domain containing 91 [Source:HGNC Symbol;Acc:HGNC:24855] | 1751 | 0.392 | 0.2974 | Yes |
| 52 | AP4M1 | adaptor related protein complex 4 subunit mu 1 [Source:HGNC Symbol;Acc:HGNC:574] | 1756 | 0.390 | 0.3024 | Yes |
| 53 | PEF1 | penta-EF-hand domain containing 1 [Source:HGNC Symbol;Acc:HGNC:30009] | 1784 | 0.387 | 0.3055 | Yes |
| 54 | TRAPPC12 | trafficking protein particle complex subunit 12 [Source:HGNC Symbol;Acc:HGNC:24284] | 1930 | 0.369 | 0.2991 | Yes |
| 55 | COPA | COPI coat complex subunit alpha [Source:HGNC Symbol;Acc:HGNC:2230] | 1959 | 0.366 | 0.3019 | Yes |
| 56 | ARF5 | ADP ribosylation factor 5 [Source:HGNC Symbol;Acc:HGNC:658] | 2027 | 0.358 | 0.3015 | Yes |
| 57 | SCAMP2 | secretory carrier membrane protein 2 [Source:HGNC Symbol;Acc:HGNC:10564] | 2037 | 0.357 | 0.3056 | Yes |
| 58 | ANK1 | ankyrin 1 [Source:HGNC Symbol;Acc:HGNC:492] | 2052 | 0.356 | 0.3093 | Yes |
| 59 | TMED1 | transmembrane p24 trafficking protein 1 [Source:HGNC Symbol;Acc:HGNC:17291] | 2212 | 0.337 | 0.3014 | Yes |
| 60 | KDELR1 | KDEL endoplasmic reticulum protein retention receptor 1 [Source:HGNC Symbol;Acc:HGNC:6304] | 2227 | 0.336 | 0.3048 | Yes |
| 61 | EXOC2 | exocyst complex component 2 [Source:HGNC Symbol;Acc:HGNC:24968] | 2230 | 0.336 | 0.3092 | Yes |
| 62 | RAB31 | "RAB31, member RAS oncogene family [Source:HGNC Symbol;Acc:HGNC:9771]" | 2232 | 0.335 | 0.3137 | Yes |
| 63 | EXOC5 | exocyst complex component 5 [Source:HGNC Symbol;Acc:HGNC:10696] | 2243 | 0.334 | 0.3174 | Yes |
| 64 | TMED7 | transmembrane p24 trafficking protein 7 [Source:HGNC Symbol;Acc:HGNC:24253] | 2256 | 0.331 | 0.3210 | Yes |
| 65 | GOLGA7 | golgin A7 [Source:HGNC Symbol;Acc:HGNC:24876] | 2370 | 0.318 | 0.3164 | Yes |
| 66 | RAB1B | "RAB1B, member RAS oncogene family [Source:HGNC Symbol;Acc:HGNC:18370]" | 2453 | 0.308 | 0.3141 | Yes |
| 67 | CTAGE9 | CTAGE family member 9 [Source:HGNC Symbol;Acc:HGNC:37275] | 2468 | 0.307 | 0.3172 | Yes |
| 68 | RABIF | RAB interacting factor [Source:HGNC Symbol;Acc:HGNC:9797] | 2488 | 0.304 | 0.3198 | Yes |
| 69 | TRAPPC3 | trafficking protein particle complex subunit 3 [Source:HGNC Symbol;Acc:HGNC:19942] | 2527 | 0.300 | 0.3209 | Yes |
| 70 | ARFGAP2 | ADP ribosylation factor GTPase activating protein 2 [Source:HGNC Symbol;Acc:HGNC:13504] | 2548 | 0.297 | 0.3233 | Yes |
| 71 | TMED9 | transmembrane p24 trafficking protein 9 [Source:HGNC Symbol;Acc:HGNC:24878] | 2600 | 0.290 | 0.3233 | Yes |
| 72 | RAB14 | "RAB14, member RAS oncogene family [Source:HGNC Symbol;Acc:HGNC:16524]" | 2601 | 0.290 | 0.3272 | Yes |
| 73 | DOP1B | DOP1 leucine zipper like protein B [Source:HGNC Symbol;Acc:HGNC:1291] | 2687 | 0.280 | 0.3243 | Yes |
| 74 | TMEM115 | transmembrane protein 115 [Source:HGNC Symbol;Acc:HGNC:30055] | 2735 | 0.276 | 0.3244 | Yes |
| 75 | NAPA | NSF attachment protein alpha [Source:HGNC Symbol;Acc:HGNC:7641] | 2742 | 0.276 | 0.3276 | Yes |
| 76 | SAR1B | secretion associated Ras related GTPase 1B [Source:HGNC Symbol;Acc:HGNC:10535] | 2780 | 0.272 | 0.3284 | Yes |
| 77 | STX6 | syntaxin 6 [Source:HGNC Symbol;Acc:HGNC:11441] | 2863 | 0.264 | 0.3255 | No |
| 78 | SNX1 | sorting nexin 1 [Source:HGNC Symbol;Acc:HGNC:11172] | 2906 | 0.260 | 0.3257 | No |
| 79 | SCFD1 | sec1 family domain containing 1 [Source:HGNC Symbol;Acc:HGNC:20726] | 2935 | 0.257 | 0.3270 | No |
| 80 | RAB29 | "RAB29, member RAS oncogene family [Source:HGNC Symbol;Acc:HGNC:9789]" | 3020 | 0.247 | 0.3237 | No |
| 81 | MYO5A | myosin VA [Source:HGNC Symbol;Acc:HGNC:7602] | 3330 | 0.218 | 0.3023 | No |
| 82 | VAMP7 | vesicle associated membrane protein 7 [Source:HGNC Symbol;Acc:HGNC:11486] | 3380 | 0.214 | 0.3013 | No |
| 83 | ATP9A | ATPase phospholipid transporting 9A (putative) [Source:HGNC Symbol;Acc:HGNC:13540] | 3398 | 0.212 | 0.3029 | No |
| 84 | SNX3 | sorting nexin 3 [Source:HGNC Symbol;Acc:HGNC:11174] | 3469 | 0.205 | 0.3001 | No |
| 85 | AP3S1 | adaptor related protein complex 3 subunit sigma 1 [Source:HGNC Symbol;Acc:HGNC:2013] | 3479 | 0.204 | 0.3022 | No |
| 86 | CUL3 | cullin 3 [Source:HGNC Symbol;Acc:HGNC:2553] | 3480 | 0.204 | 0.3050 | No |
| 87 | SORT1 | sortilin 1 [Source:HGNC Symbol;Acc:HGNC:11186] | 3481 | 0.204 | 0.3078 | No |
| 88 | VCP | valosin containing protein [Source:HGNC Symbol;Acc:HGNC:12666] | 3518 | 0.201 | 0.3076 | No |
| 89 | MIA3 | MIA SH3 domain ER export factor 3 [Source:HGNC Symbol;Acc:HGNC:24008] | 3550 | 0.198 | 0.3079 | No |
| 90 | KLHL12 | kelch like family member 12 [Source:HGNC Symbol;Acc:HGNC:19360] | 3609 | 0.193 | 0.3059 | No |
| 91 | ZW10 | zw10 kinetochore protein [Source:HGNC Symbol;Acc:HGNC:13194] | 3643 | 0.190 | 0.3059 | No |
| 92 | RAB33B | "RAB33B, member RAS oncogene family [Source:HGNC Symbol;Acc:HGNC:16075]" | 3673 | 0.188 | 0.3062 | No |
| 93 | SCAMP1 | secretory carrier membrane protein 1 [Source:HGNC Symbol;Acc:HGNC:10563] | 3682 | 0.187 | 0.3081 | No |
| 94 | TFG | trafficking from ER to golgi regulator [Source:HGNC Symbol;Acc:HGNC:11758] | 3719 | 0.183 | 0.3077 | No |
| 95 | GAS1 | growth arrest specific 1 [Source:HGNC Symbol;Acc:HGNC:4165] | 3757 | 0.181 | 0.3073 | No |
| 96 | COG2 | component of oligomeric golgi complex 2 [Source:HGNC Symbol;Acc:HGNC:6546] | 3767 | 0.180 | 0.3090 | No |
| 97 | SEC23B | "SEC23 homolog B, COPII coat complex component [Source:HGNC Symbol;Acc:HGNC:10702]" | 3838 | 0.172 | 0.3058 | No |
| 98 | SEC23IP | SEC23 interacting protein [Source:HGNC Symbol;Acc:HGNC:17018] | 3889 | 0.166 | 0.3041 | No |
| 99 | VAPB | VAMP associated protein B and C [Source:HGNC Symbol;Acc:HGNC:12649] | 3905 | 0.164 | 0.3052 | No |
| 100 | VPS52 | VPS52 subunit of GARP complex [Source:HGNC Symbol;Acc:HGNC:10518] | 3908 | 0.164 | 0.3072 | No |
| 101 | ANK3 | ankyrin 3 [Source:HGNC Symbol;Acc:HGNC:494] | 3921 | 0.163 | 0.3085 | No |
| 102 | SEC24B | "SEC24 homolog B, COPII coat complex component [Source:HGNC Symbol;Acc:HGNC:10704]" | 3933 | 0.162 | 0.3098 | No |
| 103 | VAMP5 | vesicle associated membrane protein 5 [Source:HGNC Symbol;Acc:HGNC:12646] | 3935 | 0.162 | 0.3119 | No |
| 104 | SEC23A | "SEC23 homolog A, COPII coat complex component [Source:HGNC Symbol;Acc:HGNC:10701]" | 4017 | 0.154 | 0.3076 | No |
| 105 | ATL2 | atlastin GTPase 2 [Source:HGNC Symbol;Acc:HGNC:24047] | 4025 | 0.154 | 0.3092 | No |
| 106 | PRKD1 | protein kinase D1 [Source:HGNC Symbol;Acc:HGNC:9407] | 4123 | 0.144 | 0.3035 | No |
| 107 | ERGIC2 | ERGIC and golgi 2 [Source:HGNC Symbol;Acc:HGNC:30208] | 4126 | 0.144 | 0.3053 | No |
| 108 | WIPI1 | "WD repeat domain, phosphoinositide interacting 1 [Source:HGNC Symbol;Acc:HGNC:25471]" | 4188 | 0.139 | 0.3023 | No |
| 109 | AP3D1 | adaptor related protein complex 3 subunit delta 1 [Source:HGNC Symbol;Acc:HGNC:568] | 4198 | 0.138 | 0.3035 | No |
| 110 | YIPF4 | Yip1 domain family member 4 [Source:HGNC Symbol;Acc:HGNC:28145] | 4252 | 0.133 | 0.3011 | No |
| 111 | STXBP6 | syntaxin binding protein 6 [Source:HGNC Symbol;Acc:HGNC:19666] | 4322 | 0.126 | 0.2974 | No |
| 112 | KIF16B | kinesin family member 16B [Source:HGNC Symbol;Acc:HGNC:15869] | 4355 | 0.124 | 0.2965 | No |
| 113 | EHD3 | EH domain containing 3 [Source:HGNC Symbol;Acc:HGNC:3244] | 4395 | 0.121 | 0.2951 | No |
| 114 | GOLPH3 | golgi phosphoprotein 3 [Source:HGNC Symbol;Acc:HGNC:15452] | 4420 | 0.119 | 0.2948 | No |
| 115 | NRBP1 | nuclear receptor binding protein 1 [Source:HGNC Symbol;Acc:HGNC:7993] | 4423 | 0.119 | 0.2963 | No |
| 116 | MYO1B | myosin IB [Source:HGNC Symbol;Acc:HGNC:7596] | 4425 | 0.119 | 0.2978 | No |
| 117 | EXOC6B | exocyst complex component 6B [Source:HGNC Symbol;Acc:HGNC:17085] | 4492 | 0.114 | 0.2942 | No |
| 118 | TAPBP | TAP binding protein [Source:HGNC Symbol;Acc:HGNC:11566] | 4527 | 0.111 | 0.2930 | No |
| 119 | CSNK1D | casein kinase 1 delta [Source:HGNC Symbol;Acc:HGNC:2452] | 4529 | 0.111 | 0.2944 | No |
| 120 | CTAGE1 | cutaneous T cell lymphoma-associated antigen 1 [Source:HGNC Symbol;Acc:HGNC:24346] | 4530 | 0.111 | 0.2959 | No |
| 121 | ATP9B | ATPase phospholipid transporting 9B (putative) [Source:HGNC Symbol;Acc:HGNC:13541] | 4606 | 0.104 | 0.2914 | No |
| 122 | GOLGA4 | golgin A4 [Source:HGNC Symbol;Acc:HGNC:4427] | 4667 | 0.099 | 0.2880 | No |
| 123 | GGA2 | "golgi associated, gamma adaptin ear containing, ARF binding protein 2 [Source:HGNC Symbol;Acc:HGNC:16064]" | 4671 | 0.098 | 0.2891 | No |
| 124 | CCDC93 | coiled-coil domain containing 93 [Source:HGNC Symbol;Acc:HGNC:25611] | 4732 | 0.093 | 0.2857 | No |
| 125 | STX18 | syntaxin 18 [Source:HGNC Symbol;Acc:HGNC:15942] | 4968 | 0.075 | 0.2681 | No |
| 126 | USO1 | USO1 vesicle transport factor [Source:HGNC Symbol;Acc:HGNC:30904] | 4992 | 0.074 | 0.2673 | No |
| 127 | VAMP3 | vesicle associated membrane protein 3 [Source:HGNC Symbol;Acc:HGNC:12644] | 5028 | 0.071 | 0.2655 | No |
| 128 | GOSR2 | golgi SNAP receptor complex member 2 [Source:HGNC Symbol;Acc:HGNC:4431] | 5037 | 0.070 | 0.2658 | No |
| 129 | VPS35L | VPS35 endosomal protein sorting factor like [Source:HGNC Symbol;Acc:HGNC:24641] | 5067 | 0.067 | 0.2644 | No |
| 130 | TRIP11 | thyroid hormone receptor interactor 11 [Source:HGNC Symbol;Acc:HGNC:12305] | 5233 | 0.054 | 0.2521 | No |
| 131 | SCAMP3 | secretory carrier membrane protein 3 [Source:HGNC Symbol;Acc:HGNC:10565] | 5245 | 0.052 | 0.2520 | No |
| 132 | DOP1A | DOP1 leucine zipper like protein A [Source:HGNC Symbol;Acc:HGNC:21194] | 5258 | 0.051 | 0.2517 | No |
| 133 | OPTN | optineurin [Source:HGNC Symbol;Acc:HGNC:17142] | 5323 | 0.046 | 0.2473 | No |
| 134 | CCDC22 | coiled-coil domain containing 22 [Source:HGNC Symbol;Acc:HGNC:28909] | 5493 | 0.033 | 0.2344 | No |
| 135 | RNF139 | ring finger protein 139 [Source:HGNC Symbol;Acc:HGNC:17023] | 5636 | 0.021 | 0.2235 | No |
| 136 | TMED2 | transmembrane p24 trafficking protein 2 [Source:HGNC Symbol;Acc:HGNC:16996] | 5709 | 0.015 | 0.2180 | No |
| 137 | STX10 | syntaxin 10 [Source:HGNC Symbol;Acc:HGNC:11428] | 5726 | 0.013 | 0.2169 | No |
| 138 | VPS13A | vacuolar protein sorting 13 homolog A [Source:HGNC Symbol;Acc:HGNC:1908] | 5793 | 0.009 | 0.2118 | No |
| 139 | YKT6 | YKT6 v-SNARE homolog [Source:HGNC Symbol;Acc:HGNC:16959] | 5800 | 0.008 | 0.2115 | No |
| 140 | RINT1 | RAD50 interactor 1 [Source:HGNC Symbol;Acc:HGNC:21876] | 5863 | 0.004 | 0.2066 | No |
| 141 | RACK1 | receptor for activated C kinase 1 [Source:HGNC Symbol;Acc:HGNC:4399] | 5983 | -0.003 | 0.1973 | No |
| 142 | SEC31A | "SEC31 homolog A, COPII coat complex component [Source:HGNC Symbol;Acc:HGNC:17052]" | 6021 | -0.006 | 0.1944 | No |
| 143 | PREB | prolactin regulatory element binding [Source:HGNC Symbol;Acc:HGNC:9356] | 6044 | -0.008 | 0.1928 | No |
| 144 | SORL1 | sortilin related receptor 1 [Source:HGNC Symbol;Acc:HGNC:11185] | 6143 | -0.016 | 0.1853 | No |
| 145 | GOSR1 | golgi SNAP receptor complex member 1 [Source:HGNC Symbol;Acc:HGNC:4430] | 6166 | -0.018 | 0.1838 | No |
| 146 | PPP6C | protein phosphatase 6 catalytic subunit [Source:HGNC Symbol;Acc:HGNC:9323] | 6195 | -0.020 | 0.1818 | No |
| 147 | ARFGEF2 | ADP ribosylation factor guanine nucleotide exchange factor 2 [Source:HGNC Symbol;Acc:HGNC:15853] | 6333 | -0.031 | 0.1714 | No |
| 148 | SEC24D | "SEC24 homolog D, COPII coat complex component [Source:HGNC Symbol;Acc:HGNC:10706]" | 6335 | -0.031 | 0.1718 | No |
| 149 | LMAN1 | "lectin, mannose binding 1 [Source:HGNC Symbol;Acc:HGNC:6631]" | 6361 | -0.032 | 0.1703 | No |
| 150 | SEC24C | "SEC24 homolog C, COPII coat complex component [Source:HGNC Symbol;Acc:HGNC:10705]" | 6366 | -0.033 | 0.1704 | No |
| 151 | TRAPPC11 | trafficking protein particle complex subunit 11 [Source:HGNC Symbol;Acc:HGNC:25751] | 6423 | -0.037 | 0.1665 | No |
| 152 | LLGL1 | LLGL scribble cell polarity complex component 1 [Source:HGNC Symbol;Acc:HGNC:6628] | 6528 | -0.044 | 0.1588 | No |
| 153 | HYOU1 | hypoxia up-regulated 1 [Source:HGNC Symbol;Acc:HGNC:16931] | 6775 | -0.061 | 0.1403 | No |
| 154 | ARF4 | ADP ribosylation factor 4 [Source:HGNC Symbol;Acc:HGNC:655] | 6894 | -0.072 | 0.1319 | No |
| 155 | STX5 | syntaxin 5 [Source:HGNC Symbol;Acc:HGNC:11440] | 6932 | -0.074 | 0.1300 | No |
| 156 | VPS54 | VPS54 subunit of GARP complex [Source:HGNC Symbol;Acc:HGNC:18652] | 7021 | -0.082 | 0.1242 | No |
| 157 | KRT18 | keratin 18 [Source:HGNC Symbol;Acc:HGNC:6430] | 7027 | -0.082 | 0.1249 | No |
| 158 | EXOC1 | exocyst complex component 1 [Source:HGNC Symbol;Acc:HGNC:30380] | 7102 | -0.088 | 0.1202 | No |
| 159 | MON2 | "MON2 homolog, regulator of endosome-to-Golgi trafficking [Source:HGNC Symbol;Acc:HGNC:29177]" | 7133 | -0.091 | 0.1191 | No |
| 160 | LAPTM5 | lysosomal protein transmembrane 5 [Source:HGNC Symbol;Acc:HGNC:29612] | 7168 | -0.093 | 0.1177 | No |
| 161 | GCC2 | GRIP and coiled-coil domain containing 2 [Source:HGNC Symbol;Acc:HGNC:23218] | 7280 | -0.101 | 0.1103 | No |
| 162 | CORO7 | coronin 7 [Source:HGNC Symbol;Acc:HGNC:26161] | 7283 | -0.101 | 0.1115 | No |
| 163 | BLZF1 | basic leucine zipper nuclear factor 1 [Source:HGNC Symbol;Acc:HGNC:1065] | 7289 | -0.102 | 0.1125 | No |
| 164 | AP1G2 | adaptor related protein complex 1 subunit gamma 2 [Source:HGNC Symbol;Acc:HGNC:556] | 7305 | -0.103 | 0.1127 | No |
| 165 | RAB26 | "RAB26, member RAS oncogene family [Source:HGNC Symbol;Acc:HGNC:14259]" | 7390 | -0.109 | 0.1075 | No |
| 166 | SNAP23 | synaptosome associated protein 23 [Source:HGNC Symbol;Acc:HGNC:11131] | 7547 | -0.122 | 0.0969 | No |
| 167 | ARFRP1 | ADP ribosylation factor related protein 1 [Source:HGNC Symbol;Acc:HGNC:662] | 7574 | -0.125 | 0.0965 | No |
| 168 | CREB3L2 | cAMP responsive element binding protein 3 like 2 [Source:HGNC Symbol;Acc:HGNC:23720] | 7580 | -0.125 | 0.0978 | No |
| 169 | TMED5 | transmembrane p24 trafficking protein 5 [Source:HGNC Symbol;Acc:HGNC:24251] | 7581 | -0.125 | 0.0995 | No |
| 170 | VAMP4 | vesicle associated membrane protein 4 [Source:HGNC Symbol;Acc:HGNC:12645] | 7608 | -0.128 | 0.0992 | No |
| 171 | HTT | huntingtin [Source:HGNC Symbol;Acc:HGNC:4851] | 7641 | -0.130 | 0.0985 | No |
| 172 | SCAP | SREBF chaperone [Source:HGNC Symbol;Acc:HGNC:30634] | 7647 | -0.130 | 0.0998 | No |
| 173 | GGA3 | "golgi associated, gamma adaptin ear containing, ARF binding protein 3 [Source:HGNC Symbol;Acc:HGNC:17079]" | 8022 | -0.157 | 0.0724 | No |
| 174 | CLN3 | "CLN3 lysosomal/endosomal transmembrane protein, battenin [Source:HGNC Symbol;Acc:HGNC:2074]" | 8094 | -0.163 | 0.0691 | No |
| 175 | GGA1 | "golgi associated, gamma adaptin ear containing, ARF binding protein 1 [Source:HGNC Symbol;Acc:HGNC:17842]" | 8106 | -0.163 | 0.0704 | No |
| 176 | ARCN1 | archain 1 [Source:HGNC Symbol;Acc:HGNC:649] | 8112 | -0.164 | 0.0722 | No |
| 177 | RABEP1 | "rabaptin, RAB GTPase binding effector protein 1 [Source:HGNC Symbol;Acc:HGNC:17677]" | 8165 | -0.168 | 0.0704 | No |
| 178 | ACSL3 | acyl-CoA synthetase long chain family member 3 [Source:HGNC Symbol;Acc:HGNC:3570] | 8170 | -0.169 | 0.0724 | No |
| 179 | BICD2 | BICD cargo adaptor 2 [Source:HGNC Symbol;Acc:HGNC:17208] | 8253 | -0.175 | 0.0683 | No |
| 180 | COPG1 | COPI coat complex subunit gamma 1 [Source:HGNC Symbol;Acc:HGNC:2236] | 8289 | -0.178 | 0.0679 | No |
| 181 | RAB6B | "RAB6B, member RAS oncogene family [Source:HGNC Symbol;Acc:HGNC:14902]" | 8309 | -0.179 | 0.0689 | No |
| 182 | SPTBN1 | "spectrin beta, non-erythrocytic 1 [Source:HGNC Symbol;Acc:HGNC:11275]" | 8335 | -0.181 | 0.0694 | No |
| 183 | WHAMMP3 | "WAS protein homolog associated with actin, golgi membranes and microtubules pseudogene 3 [Source:HGNC Symbol;Acc:HGNC:27892]" | 8350 | -0.182 | 0.0707 | No |
| 184 | CNIH4 | cornichon family AMPA receptor auxiliary protein 4 [Source:HGNC Symbol;Acc:HGNC:25013] | 8392 | -0.185 | 0.0700 | No |
| 185 | GBF1 | golgi brefeldin A resistant guanine nucleotide exchange factor 1 [Source:HGNC Symbol;Acc:HGNC:4181] | 8461 | -0.191 | 0.0672 | No |
| 186 | STX17 | syntaxin 17 [Source:HGNC Symbol;Acc:HGNC:11432] | 8466 | -0.191 | 0.0695 | No |
| 187 | UVRAG | UV radiation resistance associated [Source:HGNC Symbol;Acc:HGNC:12640] | 8555 | -0.198 | 0.0652 | No |
| 188 | PHAF1 | phagosome assembly factor 1 [Source:HGNC Symbol;Acc:HGNC:29564] | 8600 | -0.201 | 0.0645 | No |
| 189 | SEC22B | "SEC22 homolog B, vesicle trafficking protein [Source:HGNC Symbol;Acc:HGNC:10700]" | 8614 | -0.202 | 0.0662 | No |
| 190 | VAPA | VAMP associated protein A [Source:HGNC Symbol;Acc:HGNC:12648] | 8854 | -0.221 | 0.0504 | No |
| 191 | VAMP2 | vesicle associated membrane protein 2 [Source:HGNC Symbol;Acc:HGNC:12643] | 8932 | -0.226 | 0.0474 | No |
| 192 | LYPLA1 | lysophospholipase 1 [Source:HGNC Symbol;Acc:HGNC:6737] | 9023 | -0.234 | 0.0434 | No |
| 193 | CSK | C-terminal Src kinase [Source:HGNC Symbol;Acc:HGNC:2444] | 9056 | -0.236 | 0.0441 | No |
| 194 | ANKFY1 | ankyrin repeat and FYVE domain containing 1 [Source:HGNC Symbol;Acc:HGNC:20763] | 9061 | -0.237 | 0.0470 | No |
| 195 | MACF1 | microtubule actin crosslinking factor 1 [Source:HGNC Symbol;Acc:HGNC:13664] | 9123 | -0.241 | 0.0455 | No |
| 196 | SEC24A | "SEC24 homolog A, COPII coat complex component [Source:HGNC Symbol;Acc:HGNC:10703]" | 9357 | -0.260 | 0.0306 | No |
| 197 | PGAP1 | post-GPI attachment to proteins inositol deacylase 1 [Source:HGNC Symbol;Acc:HGNC:25712] | 9358 | -0.260 | 0.0341 | No |
| 198 | AP1G1 | adaptor related protein complex 1 subunit gamma 1 [Source:HGNC Symbol;Acc:HGNC:555] | 9389 | -0.263 | 0.0354 | No |
| 199 | EPS15 | epidermal growth factor receptor pathway substrate 15 [Source:HGNC Symbol;Acc:HGNC:3419] | 9409 | -0.265 | 0.0375 | No |
| 200 | SEC16A | "SEC16 homolog A, endoplasmic reticulum export factor [Source:HGNC Symbol;Acc:HGNC:29006]" | 9434 | -0.268 | 0.0392 | No |
| 201 | COPB1 | COPI coat complex subunit beta 1 [Source:HGNC Symbol;Acc:HGNC:2231] | 9447 | -0.269 | 0.0419 | No |
| 202 | STEEP1 | STING1 ER exit protein 1 [Source:HGNC Symbol;Acc:HGNC:26239] | 9592 | -0.282 | 0.0344 | No |
| 203 | RAB1A | "RAB1A, member RAS oncogene family [Source:HGNC Symbol;Acc:HGNC:9758]" | 9604 | -0.284 | 0.0374 | No |
| 204 | RSC1A1 | regulator of solute carriers 1 [Source:HGNC Symbol;Acc:HGNC:10458] | 9664 | -0.288 | 0.0366 | No |
| 205 | CTAGE6 | CTAGE family member 6 [Source:HGNC Symbol;Acc:HGNC:28644] | 9691 | -0.290 | 0.0385 | No |
| 206 | PLPP3 | phospholipid phosphatase 3 [Source:HGNC Symbol;Acc:HGNC:9229] | 9906 | -0.312 | 0.0259 | No |
| 207 | KIF1C | kinesin family member 1C [Source:HGNC Symbol;Acc:HGNC:6317] | 9942 | -0.316 | 0.0274 | No |
| 208 | YIF1B | "Yip1 interacting factor homolog B, membrane trafficking protein [Source:HGNC Symbol;Acc:HGNC:30511]" | 9960 | -0.317 | 0.0303 | No |
| 209 | TMED10 | transmembrane p24 trafficking protein 10 [Source:HGNC Symbol;Acc:HGNC:16998] | 9993 | -0.320 | 0.0322 | No |
| 210 | RAB8B | "RAB8B, member RAS oncogene family [Source:HGNC Symbol;Acc:HGNC:30273]" | 10223 | -0.343 | 0.0188 | No |
| 211 | INSIG1 | insulin induced gene 1 [Source:HGNC Symbol;Acc:HGNC:6083] | 10264 | -0.347 | 0.0203 | No |
| 212 | GOLGA2 | golgin A2 [Source:HGNC Symbol;Acc:HGNC:4425] | 10285 | -0.349 | 0.0235 | No |
| 213 | GAK | cyclin G associated kinase [Source:HGNC Symbol;Acc:HGNC:4113] | 10301 | -0.350 | 0.0271 | No |
| 214 | RP2 | RP2 activator of ARL3 GTPase [Source:HGNC Symbol;Acc:HGNC:10274] | 10354 | -0.356 | 0.0278 | No |
| 215 | ARFGAP3 | ADP ribosylation factor GTPase activating protein 3 [Source:HGNC Symbol;Acc:HGNC:661] | 10382 | -0.359 | 0.0305 | No |
| 216 | SGSM2 | small G protein signaling modulator 2 [Source:HGNC Symbol;Acc:HGNC:29026] | 10403 | -0.361 | 0.0339 | No |
| 217 | ATP2C1 | ATPase secretory pathway Ca2+ transporting 1 [Source:HGNC Symbol;Acc:HGNC:13211] | 10432 | -0.364 | 0.0366 | No |
| 218 | TRAPPC8 | trafficking protein particle complex subunit 8 [Source:HGNC Symbol;Acc:HGNC:29169] | 10542 | -0.377 | 0.0331 | No |
| 219 | NAPG | NSF attachment protein gamma [Source:HGNC Symbol;Acc:HGNC:7642] | 10671 | -0.390 | 0.0283 | No |
| 220 | RAB13 | "RAB13, member RAS oncogene family [Source:HGNC Symbol;Acc:HGNC:9762]" | 10961 | -0.425 | 0.0113 | No |
| 221 | LAMP1 | lysosomal associated membrane protein 1 [Source:HGNC Symbol;Acc:HGNC:6499] | 11142 | -0.449 | 0.0032 | No |
| 222 | PICK1 | protein interacting with PRKCA 1 [Source:HGNC Symbol;Acc:HGNC:9394] | 11234 | -0.461 | 0.0023 | No |
| 223 | LMAN1L | "lectin, mannose binding 1 like [Source:HGNC Symbol;Acc:HGNC:6632]" | 11430 | -0.492 | -0.0064 | No |
| 224 | PITPNB | phosphatidylinositol transfer protein beta [Source:HGNC Symbol;Acc:HGNC:9002] | 11527 | -0.505 | -0.0072 | No |
| 225 | KLHL20 | kelch like family member 20 [Source:HGNC Symbol;Acc:HGNC:25056] | 11565 | -0.510 | -0.0031 | No |
| 226 | MIA2 | MIA SH3 domain ER export factor 2 [Source:HGNC Symbol;Acc:HGNC:18432] | 11652 | -0.525 | -0.0028 | No |
| 227 | SEC31B | "SEC31 homolog B, COPII coat complex component [Source:HGNC Symbol;Acc:HGNC:23197]" | 11690 | -0.531 | 0.0015 | No |
| 228 | TRAPPC10 | trafficking protein particle complex subunit 10 [Source:HGNC Symbol;Acc:HGNC:11868] | 11818 | -0.553 | -0.0010 | No |
| 229 | SAR1A | secretion associated Ras related GTPase 1A [Source:HGNC Symbol;Acc:HGNC:10534] | 11962 | -0.586 | -0.0043 | No |
| 230 | BET1L | Bet1 golgi vesicular membrane trafficking protein like [Source:HGNC Symbol;Acc:HGNC:19348] | 12084 | -0.615 | -0.0055 | No |
| 231 | AMN | amnion associated transmembrane protein [Source:HGNC Symbol;Acc:HGNC:14604] | 12281 | -0.671 | -0.0119 | No |
| 232 | AP1AR | adaptor related protein complex 1 associated regulatory protein [Source:HGNC Symbol;Acc:HGNC:28808] | 12323 | -0.683 | -0.0059 | No |
| 233 | DNM2 | dynamin 2 [Source:HGNC Symbol;Acc:HGNC:2974] | 12362 | -0.695 | 0.0006 | No |
| 234 | VPS13C | vacuolar protein sorting 13 homolog C [Source:HGNC Symbol;Acc:HGNC:23594] | 12367 | -0.696 | 0.0097 | No |
| 235 | PRKCI | protein kinase C iota [Source:HGNC Symbol;Acc:HGNC:9404] | 12444 | -0.723 | 0.0136 | No |
| 236 | CYTH3 | cytohesin 3 [Source:HGNC Symbol;Acc:HGNC:9504] | 12632 | -0.814 | 0.0099 | No |
| 237 | KIF13A | kinesin family member 13A [Source:HGNC Symbol;Acc:HGNC:14566] | 12650 | -0.826 | 0.0197 | No |
Table: GSEA details [plain text format]

  

Fig 2: GOBP\_GOLGI\_VESICLE\_TRANSPORT      
 Blue-Pink O' Gram in the Space of the Analyzed GeneSet

  

Fig 3: GOBP\_GOLGI\_VESICLE\_TRANSPORT: Random ES distribution      
 Gene set null distribution of ES for **GOBP\_GOLGI\_VESICLE\_TRANSPORT**
